# The effect of early activity on postoperative patients with lung cancer: a systematic review and meta-analysis

**DOI:** 10.3389/fonc.2026.1840866

**Published:** 2026-08-20

**Authors:** Zengxu Wang, Yanli Xiu, Xiaoxuan Qi, Mengxian Wang, Qi Li, Lingjia Cui, Huiying Yue, Feng Liu, Ying Lin

**Affiliations:** 1 Department of Nursing, Sir Run Run Shaw Hospital, School of Medicine, Zhejiang University, Hangzhou, China; 2 Department of Thoracic Surgery, Hongqi Hospital Affiliated to Mudanjiang Medical University, Mudanjiang, China; 3 School of Nursing, Mudanjiang Medical University, Mudanjiang, China

**Keywords:** early activity, lung cancer, meta-analysis, postoperative patients, systematic review

## Abstract

**Background:**

Early activity is recognized as a safe and effective therapeutic approach that offers benefits in accelerating the recovery of patients with lung cancer after surgery.

**Objectives:**

Data from studies that met the inclusion criteria were pooled to assess the impact of early activity on the prognosis of postoperative lung cancer patients, including duration of chest tube retention, length of hospital stay (LOS), incidence of complications, lung function, and 6-minute walking distance (6MWD).

**Methods:**

Electronic databases, including Web of Science, CINAHL, the Cochrane Library, EMBASE, PubMed, the Chinese BioMedical Literature Service System (SinoMed), WANFANG, CNKI, VIP databases, and Clinical Trial Register Platform were systematically searched from inception to March 11, 2026. Two researchers independently assessed study eligibility. Titles and abstracts were initially screened, followed by a full-text review of potentially eligible articles. Data extraction and risk of bias (RoB) assessment were performed for all studies meeting the eligibility criteria. Pooled outcomes were presented as mean differences (MDs) or risk ratios (RRs) with corresponding 95% confidence intervals (CIs).

**Results:**

Twenty-three studies involving 2,234 participants were included in the meta-analysis. The results demonstrated that early activity significantly improved postoperative outcomes in patients with lung cancer. It notably reduced the incidence of pulmonary complications, LOS, and duration of chest tube retention. Subgroup analysis of lung function outcomes showed that early activity significantly improved forced expiratory volume in the first second (FEV1), whereas no significant effect was observed for FEV1%. Regarding exercise capacity, early in-hospital activity alone significantly improved 6MWD; however, combining it with post-discharge programs yielded no significant benefit and showed substantial heterogeneity.

**Conclusions:**

This study’s findings underscore the significant role of early activity in the recovery of postoperative patients with lung cancer. While early in-hospital activity effectively reduced complications, chest tube duration, and LOS and improved FEV1, its effects on FEV1% and long-term exercise capacity remain uncertain due to inconsistent results and high heterogeneity in existing studies. Further large-scale, multicenter, randomized controlled trials (RCTs) are needed to strengthen these findings.

**Systematic Review Registration:**

https://www.crd.york.ac.uk/prospero/, identifier CRD42022333696.

## 1 Introduction

Lung cancer remains one of the most prevalent malignant tumors worldwide. According to Global Cancer Statistics 2022, it is the most frequently diagnosed cancer and ranked first in cancer-related mortality (1). For patients with early-stage lung cancer, surgery is the most effective treatment (2, 3). However, postoperative pulmonary problems, including respiratory failure, pneumonia, myocardial infarction, and arrhythmias, frequently occur (4, 5). Early postoperative symptoms such as pain and impaired respiratory function, coupled with complications like pulmonary infection and atelectasis, significantly hinder patient rehabilitation. Addressing these issues is essential to accelerate pulmonary recovery following thoracic surgery. Furthermore, lung resection surgery inherently diminishes exercise capacity (4, 6). Existing national and international enhanced recovery guidelines emphasize that early postoperative exercise reduces pulmonary complications and shortens the length of hospital stay (LOS) (7–10). Consequently, exercise training is increasingly recognized as an essential component of postoperative rehabilitation in lung cancer care.

The 2019 guidelines from the Enhanced Recovery After Surgery (ERAS) define early activity as getting out of bed within 24 hours after surgery (8). This immediate postoperative period represents a clinically important and time-sensitive window for recovery after lung cancer surgery. Mobilization within the first 24 hours may promote lung re-expansion, facilitate sputum clearance, improve circulation, reduce the risk of postoperative pulmonary complications, and support earlier chest tube removal and hospital discharge (11). Therefore, evidence specifically focused on activity initiated within this early postoperative window is important for guiding immediate postoperative nursing and rehabilitation practice.

To avoid conceptual ambiguity, we distinguished between “exercise”, “physical activity”, and “activity” in this review. Exercise generally refers to planned, structured, repetitive, and purposeful physical activity aimed at improving or maintaining physical fitness, whereas physical activity is a broader concept that includes bodily movement during daily living or rehabilitation (12). In contrast, the term “activity” or “early activity” in the present review refers to postoperative mobilization or activity-based interventions initiated within 24 hours after surgery.

In clinical practice, early activity interventions vary and may encompass respiratory training, ambulation, muscle strengthening, and aerobic exercise. Previous research indicates that exercise training improves postoperative outcomes for people with lung cancer (13). Systematic reviews of perioperative exercise therapies suggest that exercise significantly enhances functional capacity following pulmonary resection (14–17). However, findings in the literature remain inconsistent. For instance, a review conducted by Sommer et al. found that while postoperative exercise programs improved quality of life, their impact on exercise capacity remained inconclusive due to the limited scope of randomized controlled trials (RCTs) interventions (primarily strength and aerobic training) (18). Furthermore, other clinical studies have demonstrated that early activity can reduce postoperative complications (19), shorten the duration of chest drain retention (20), and improve lung function (21, 22).

Although early activity is recommended in ERAS pathways, the effectiveness of specific early activity interventions initiated within 24 hours after lung cancer surgery remains unclear (7–10). Furthermore, there is a lack of evidence synthesis specifically focusing on this immediate postoperative activity window (19–22). Therefore, this systematic review aimed to evaluate the effects of early activity—defined as structured postoperative activity initiated within 24 hours after surgery—on key postoperative recovery outcomes, including chest tube retention time, LOS, postoperative complications, pulmonary function, and exercise capacity.

## 2 Methods

The protocol for this systematic review was registered in the PROSPERO database (registration number: CRD42022333696). This review followed the methodological guidance of the Cochrane Handbook for Systematic Reviews of Interventions (Version 6.5) (23) and was reported according to the Preferred Reporting Items for Systematic Reviews and Meta-Analyses (PRISMA) guidelines (24).

### 2.1 Information sources

A comprehensive literature search was conducted from database inception to 11 March 2026 across 10 electronic databases: Web of Science, CINAHL, the Cochrane Library, EMBASE, PubMed, the Chinese BioMedical Literature Service System (CBM), WANFANG, CNKI, VIP databases, and the Clinical Trial Register Platform. To identify additional eligible studies, we manually screened the reference lists of relevant systematic reviews and included studies. Where necessary, corresponding authors were contacted via email to retrieve missing data or clarify study details.

### 2.2 Search strategy

Prior to the formal literature search, an initial scoping search was conducted to identify relevant keywords and Medical Subject Headings (MeSH) contained in the titles and abstracts of relevant articles. The search strategy was developed by the first author in collaboration with an experienced medical librarian to ensure methodological rigor. The final search utilized a combination of MeSH terms and keywords, including “early rehabilitation”, “aerobic exercise”, “respiratory training”, “early exercise”, “lung tumors”, “lung cancer”, and combinations thereof. All eligible studies were included regardless of publication status or language (**Supplementary Appendix 1**).

### 2.3 Eligibility criteria

This review included RCTs featuring at least one control or comparison group. The specific inclusion criteria were as follows:

- Participants: patients with lung cancer undergoing curative-intent surgery, regardless of gender, age, ethnicity, or country;
- Interventions: early activity interventions, defined as any structured exercise or mobilization protocol initiated within 24 hours post-surgery, encompassing early ambulation, breathing exercises, aerobic activity, and muscle strength training, either alone or in combination. To account for variability, details of the interventions were extracted;
- Comparators: control groups that did not receive early activity interventions or received only conventional postoperative care;
- Outcomes: the primary outcomes included lung function and duration of chest tube retention. Secondary outcomes included LOS, incidence of complications, and six-minute walk distance (6MWD).

Exclusion criteria included: 1) duplicate publications identified across different databases; 2) studies with unavailable, incomplete or non-comparable outcome data; 3) studies including patients with severe comorbidities or coexisting conditions that could independently affect postoperative recovery, pulmonary function, exercise capacity, or the ability to participate in early activity, such as severe cardiopulmonary disease, serious musculoskeletal or neurological disorders, other malignancies, or cognitive impairment; and 4) studies including patients who received preoperative radiotherapy, because radiotherapy may independently affect pulmonary function, postoperative complications, and recovery outcomes, thereby confounding the effect of early activity.

### 2.4 Selection process

All retrieved records from the ten databases were imported into EndNote X9 for reference management and deduplication. The titles and abstracts of all records were then screened by two independent reviewers according to predefined eligibility criteria. Full-text articles of potentially eligible studies were subsequently assessed independently by both reviewers to confirm final inclusion. Any disagreements were resolved through discussion or, if necessary, consultation with a third reviewer.

### 2.5 Data collection process and data extraction

A standardized data extraction form, adapted from the Cochrane Handbook for Systematic Reviews of Interventions, was used to guide data collection (23). The adaptations primarily involved adding specific information regarding early activity interventions, such as the timing of the first postoperative activity (in hours), the specific surgical approaches, and the incidence of postoperative complications, etc. Two reviewers independently extracted the data, and any unresolved discrepancies were addressed by consulting a third reviewer. The form was pilot-tested on two included studies and subsequently refined to better align with this review’s requirements.

### 2.6 Quality assessment

The risk of bias of the included RCTs was assessed using the Cochrane risk of bias tool 2 (25). The assessment covered five domains: bias arising from the randomization process, bias due to deviations from intended interventions, bias due to missing outcome data, bias in measurement of the outcome, and bias in selection of the reported result. Each domain was judged as “low risk of bias”, “some concerns”, or “high risk of bias” according to the RoB 2 guidance, and an overall risk-of-bias judgement was assigned for each study. Two independent reviewers evaluated the overall quality of the evidence using the Grading of Recommendations Assessment, Development, and Evaluation (GRADE) framework. The evidence was ultimately categorized as high, moderate, low, or very low quality based on an assessment of five core domains: risk of bias, inconsistency, indirectness, imprecision, and publication bias (25). Any disagreements were resolved through discussion or, if necessary, consultation with a third reviewer.

### 2.7 Synthesis methods

Meta-analysis was performed using RevMan 5.2. A narrative synthesis was adopted for studies that could not be quantitatively pooled. Dichotomous outcomes were expressed as risk ratios (RRs) with 95% confidence intervals (CIs). Continuous outcomes were reported as mean differences (MDs) when the same measurement unit was used across studies (e.g., days for length of stay, meters for walking distance), and as standardized mean differences (SMDs, Hedges’g) when different scales or units were reported (e.g., lung function measured as FEV1 or FEV1%.

For model selection, given the clinical and methodological heterogeneity among the included studies (e.g., variations in specific early activity protocols), a random-effects model was selected *a priori* to pool the effect sizes. Heterogeneity was visually assessed using forest plots and further evaluated using the Cochrane’s Q test and *I²* statistics. The degree of heterogeneity was interpreted as follows: <25% indicated no heterogeneity, 25–50% low heterogeneity, 50–75% moderate heterogeneity, and ≥75% high heterogeneity. Subgroup analyses were predefined *a priori* to explore potential sources of heterogeneity. The predefined grouping variables included the timing of early activity initiation, the specific surgical approaches, and the types of intervention. Sensitivity analysis was performed by sequentially omitting individual studies to evaluate the robustness of the pooled results. Potential publication bias was evaluated when a minimum of 10 studies were included for a specific outcome (23), utilizing a combination of visual inspection through funnel plots and Egger’s test.

## 3 Results

### 3.1 Search results

**Figure 1:**
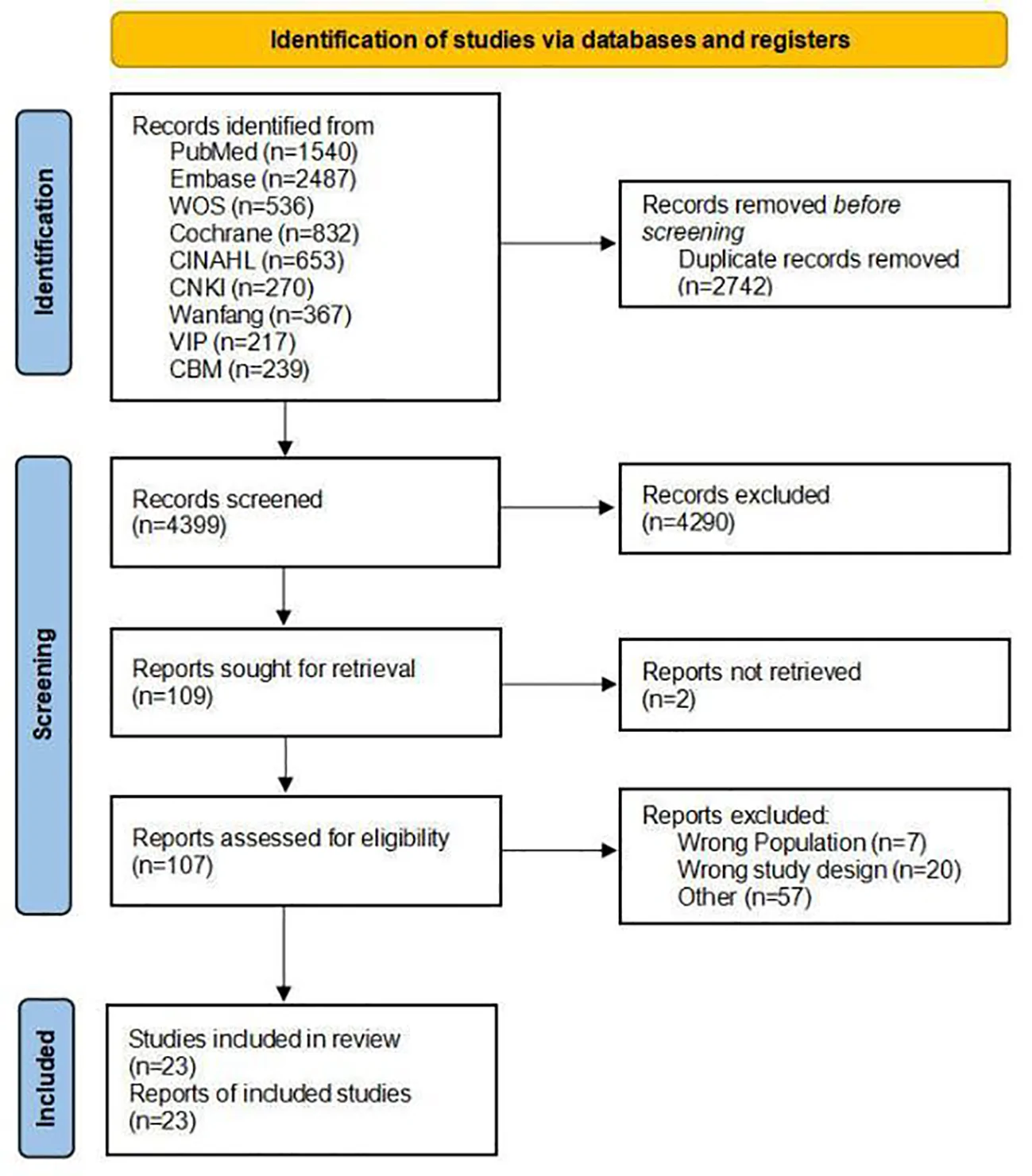
PRISMA flow chart of study selection.

The PRISMA flow chart of study selection is presented in **Figure 1**.

The initial search yielded 7,141 records from nine databases. After the removal of 2,742 duplicates, 4,399 records were remained for titles and abstracts screening, which resulted in the exclusion of 4,290 irrelevant records. Of the 109 articles assessed via full-text review, 86 were excluded: 84 due to inappropriate study design, irrelevant patient participants, or other mismatched criteria; and two due to inaccessible data. Ultimately, 23 studies met the inclusion criteria.

### 3.2 Characteristics of the included studies

The key characteristics of the 23 included studies are summarized in **Table 1**. The studies were published between 2004 and 2025 and included a total of 2,234 patients with lung cancer, with 1,119 allocated to early activity interventions and 1,115 to usual care. Most studies were conducted in China, while others were from the UK, Denmark, Australia, and Sweden. The sample sizes ranged from 15 to 300 participants. The included patients mainly underwent thoracoscopic surgery, thoracotomy, or lobectomy, although cancer stage and preoperative treatment were not consistently reported. Early activity interventions varied and included early ambulation, breathing exercises, respiratory muscle training, limb or resistance exercise. The main outcomes included chest tube duration, LOS, postoperative complications, lung function, and 6MWD.

**Table 1:** Characteristics of the included studies.

Study	Country	Sample size (I/C)	Mean age (I/C)	Male, n/% (I/C)	Cancer type/stage	Surgical approach/type	Preoperative treatment	Early activity intervention details	Comparator	Follow-up/outcomes	Key findings/notes
Arbane et al., 2011 (26)	UK	26/25 analyzed; 27/26 randomized	65.4/62.6	Overall 28/53; group-specific NR	NSCLC; stages I–III mainly	Mostly open thoracotomy or 2 VAT	NR	Twice-daily walking, marching, bedside cycling, and leg strengthening; 60%–80% max HR; plus 12-week home support	Usual care	6MWD, QoL, quadriceps strength, LOS, complications	Improved quadriceps strength; no significant effect on 6MWD, QoL.
Brocki et al., 2016 (27)	Denmark	34/34	69.7/70.5	20/19; 59%/56%	Suspected or confirmed lung tumour; NSCLC 27/25; stage NR	VATS 20/15; thoracotomy 14/19; wedge/segmentectomy, lobectomy, bilobectomy, pneumonectomy	NR	Standard physiotherapy plus IMT twice daily for 2 weeks; 2 × 30 breaths/session; target intensity 30% MIP; most inpatient sessions supervised; telephone coaching after discharge	Standard physiotherapy: breathing exercises, coughing techniques, early mobilization	MIP/MEP, 6MWT, SpO, PPCs	IMT improved oxygenation but did not significantly improve respiratory muscle strength.
Chen, 2018 (28)	China	50/50	59.3/59.7	40/38; 80%/76%	Lung cancer; stage NR	VATS radical lung cancer surgery/lobectomy	NR	ERAS nursing including preoperative education, analgesia, tube management, early feeding, and early activity. Bed exercises after awakening; sitting on POD1; standing on POD2; walking on POD3	Conventional perioperative nursing	Postoperative period; first ambulation time, chest tube duration, LOS, complications	Shortened first ambulation time, chest tube duration, and LOS; no significant reduction in pulmonary infection
Chen, 2024 (29)	China	50/50	Overall 55.72 ± 6.90	Overall 64/100 male	Stage II lung cancer	Lung cancer surgery; specific approach NR	NR	Early systematic rehabilitation: respiratory training, effective coughing/sputum clearance, limb exercises from 6–48 h post-op; increased activity on POD3–5; progressive walking and self-care after POD6	Routine nursing including psychological care, early activity encouragement, and chest tube care	Postoperative period; VC, VT, pulmonary infection, ICU/care time, LOS	Improved VC and VT; reduced pulmonary infection, monitoring time, and LOS
Deng et al., 2019 (30)	China	50/50	57.22/57.46	35/36; 70%/72%	Lung cancer; stage NR	Lung cancer surgery; specific approach NR	NR	Systematic early rehabilitation after surgery: breathing exercises, sputum clearance, limb exercises from 6–48 h; deep breathing and increased activity on POD3–5; progressive activity after POD6	Routine perioperative nursing	Postoperative period; VC, VT, pulmonary infection, monitoring time, LOS	Improved VC and VT; reduced pulmonary infection, monitoring time, and LOS
Yunting et al., 2021 (31)	China	50/50	59.0/62.65	32/36; 64%/72%	Lung cancer; TNM I–III	Thoracoscopic radical lung cancer surgery	No chemo/radiotherapy	ERAS nursing: preop education and respiratory training, multimodal analgesia, early feeding, early activity from 6 h post-op, out-of-bed after 24 h	Routine perioperative nursing	Postoperative period; pain, 6MWD, chest tube duration, LOS, pulmonary complications	Reduced pain, chest tube duration, LOS, and complications; improved 6MWD
Feng et al., 2020 (32)	China	35/35	58.6/61.4	22/20; 62.9%/57.1%	Early NSCLC; TNM I–II/part IIIA reported	FTS: single-port VATS lobectomy + lymph node dissection; control: three-port VATS	Excluded chemo/radiotherapy	FTS pathway including preop education, shortened fasting, perioperative warming/fluid control, multimodal analgesia, early oral intake, and early ambulation	Traditional perioperative treatment	Postoperative period; ambulation time, chest tube duration, pain, LOS, cost, complications	Shortened ambulation time, chest tube duration, and LOS; reduced pain and cost; no significant difference in complications
Granger et al., 2013 (22)	Australia	7/8	57.0/72.4	3/5; 42.9%/62.5%	Suspected lung cancer; postoperative lung cancer confirmed in 10/15	Lung resection; wedge resection, lobectomy, or pleurodesis	NR	Standard care plus exercise from POD1: twice-daily inpatient aerobic/resistance/stretching; home walking; outpatient supervised exercise twice weekly for 8 weeks	Standard care physiotherapy	Pre-op, 2 weeks, 12 weeks; 6MWT, TUG, HRQoL, safety/feasibility	Exercise was safe and feasible; improved 6MWT trend/significant between-group difference; no adverse events
Jonsson et al., 2019 (33)	Sweden	54/53	68.7/68.4	29/18; 53.7%/34.0%	Suspected or confirmed lung cancer; stage NR	Thoracotomy/VATS; wedge resection, lobectomy, bilobectomy, pneumonectomy	NR	In-hospital physiotherapy: early mobilization, ambulation, breathing exercises with/without PEP, thoracic/shoulder ROM; 10–30 min/session, once or twice daily	Standard care without physiotherapy	Pre-op and 3 months; 6MWT, physical activity, spirometry, dyspnea, pain	No significant between-group differences in 6MWT, physical activity, lung function, or dyspnea
Jonsson et al., 2019 (34)	Sweden	50/44	69/68	28/17; 56%/39%	Suspected or confirmed lung cancer; stage NR	Thoracotomy/VATS; wedge resection, lobectomy, bilobectomy, pneumonectomy	NR	In-hospital physiotherapy: mobilization, ambulation, shoulder exercises, and breathing exercises from early postoperative period	No physiotherapy treatment	In-hospital/POD4; accelerometer activity, 6MWT, pain, dyspnea, lung function	Increased early postoperative physical activity; no significant effect on 6MWT, spirometry, pain, or dyspnea
Quan et al., 2004 (35)	China	50/50	59/60	41/42; 82%/84%	Lung cancer; stage NR	Open thoracic lung cancer surgery	NR	Planned rehabilitation before and after surgery: breathing/cough training pre-op; from 6 h post-op breathing, limb exercises; sitting/coughing from 24–72 h; progressive walking after 72 h	Routine care and self-directed exercise	Postoperative period; complications, comfort, bed-rest time, LOS	Reduced complications, bed-rest time, and LOS; improved comfort
Shi et al., 2021 (36)	China	48/50	55.73/55.47	25/25; 52.1%/50.0%	NSCLC; stage I–III	NSCLC wedge resection	NR	Early respiratory function training from POD1–2: abdominal breathing, pursed-lip breathing, and active cycle breathing training	Routine nursing	Post-intervention; FEV1/FVC, FEV1, QoL, stress indicators	Improved FEV1/FVC and FEV1; improved QoL; reduced stress response
Wang, 2021 (37)	China	30/30	64.52/64.70	16/15; 53.3%/50.0%	Elderly lung cancer; stage NR	VATS lobectomy	NR	ERAS-based early systematic pulmonary rehabilitation, including breathing training, ACBT, respiratory muscle training, limb activity, and early ambulation	Traditional postoperative nursing	Pre-op and before discharge; FVC, FEV1, 6MWD, complications, pain, LOS	Improved FVC, FEV1, and 6MWD; reduced pain and LOS; complications not significantly different
Wang et al., 2011 (38)	China	36/38 analyzed; 40/40 randomized	62.45/64.24	25/22; 62.5%/55.0%	NSCLC; stage I–III	Radical pulmonary lobectomy; VATS/VAMT	NR	Fast-track surgery pathway including preoperative education, optimized anesthesia/analgesia, warming/fluid control, minimally invasive surgery, and early postoperative recovery measures	Conservative/traditional perioperative treatment	Postoperative period; pulmonary complications, ICU stay, LOS	Reduced pulmonary complications and LOS; ICU stay similar
Wu et al., 2022 (39)	China	30/30	51.67/52.18	13/14; 43.3%/46.7%	Lung cancer; stage I–II	VATS lobectomy	NR	Early activity program: bed exercises 4–6 h post-op, sitting/standing 6–12 h, ambulation within 24 h, daily activity and discharge education	Routine nursing	Postoperative period; chest tube duration, first defecation, first ambulation, LOS, complications	Shortened chest tube duration, first defecation time, first ambulation time, and LOS; reduced complications
Yan et al., 2020 (40)	China	78/79	60.62/61.30	35/33; 44.9%/41.8%	Lung cancer; stage NR	VATS lobectomy or sublobar resection	NR	ERAS-based early ambulation: bed activity after awakening, first ambulation at 6 h if stable, ambulation twice within 24 h, then progressive walking 2–4 times/day	Routine nursing with ambulation encouraged after 24 h	Postoperative period; drainage volume, first ambulation, first defecation, chest tube duration, LOS, pain, complications	Shortened first ambulation, first defecation, chest tube duration, and LOS; reduced pain on POD2; complications not significantly different
Yang, 2018 (41)	China	60/60	NR	NR	Lung cancer; stage NR	Lung cancer surgery; specific approach NR	NR	Systematic early rehabilitation after surgery: breathing exercise, cough/sputum training, postural drainage, and activity training	Routine nursing	Postoperative period; VC, VT, pulmonary infection, monitoring time, LOS, satisfaction	Improved VC and VT; reduced pulmonary infection, monitoring time, and LOS; higher satisfaction
Yang et al., 2020 (42)	China	63/61	52.8/52.8	35/38; 55.6%/62.3%	Lung cancer; no metastasis reported	Thoracoscopic radical lung cancer surgery	No radiotherapy or preoperative chemotherapy	FTS-CNP pathway with QR-code education: perioperative ERAS care, respiratory training, early oral intake, ankle pump/hip lifting on surgery day, assisted ambulation on POD1	Routine thoracic nursing	Postoperative period; chest tube duration, recovery days, pain, sleep, LOS, cost, complications	Shortened chest tube duration, recovery days and LOS; reduced pain, sleep score, cost and complications
Yin, 2017 (43)	China	44/44	NR	Overall 54/88 male	Stage II lung cancer	Lung cancer surgery; specific approach NR	NR	Early systematic rehabilitation from 6–48 h: breathing training, sputum clearance, limb exercises; increased breathing/limb activity and ambulation after POD3	Routine nursing	Postoperative period; VC, VT, pulmonary infection, monitoring time, LOS	Improved VC and VT; reduced pulmonary infection, monitoring time and LOS
Zhang et al., 2021 (44)	China	150/150	62.21/62.84	84/82; 56.0%/54.7%	Lung cancer; stage I–III	Lung cancer surgery; pneumonectomy excluded	No preoperative chemo/radiotherapy	ERAS-based early ambulation: preop respiratory training; bed activity after awakening; first ambulation as early as possible; ≥2 ambulations within 24 h, then progressive activity 4 times/day	Routine nursing, with ambulation encouraged after 24 h	Postoperative period; first ambulation, first flatus, chest tube duration, LOS, pain, FVC, FEV1	Shortened first ambulation, flatus, chest tube duration and LOS; reduced pain on POD2; improved FVC and FEV1
Zhu, 2021 (45)	China	35/35	68.79/68.82	27/26; 77.1%/74.3%	Lung cancer; stage NR	Lung cancer surgery; specific approach NR	NR	Systematic early rehabilitation: positioning, respiratory training, limb activity and turning on surgery day; bedside activity, ambulation, upper-limb and breathing exercises from POD1	Routine postoperative nursing	Postoperative period; first ambulation, chest tube removal, LOS, complications	Shortened first ambulation time, chest tube removal time and LOS; reduced complications
Yunting, 2025 (46)	China	45/45	68.66/68.49	31/32; 68.9%/71.1%	Lung cancer; stage NR	VATS radical lung cancer surgery	NR	Early activity nursing: preop education; passive limb activity on surgery day; respiratory training on POD1; bedside walking/ADL on POD2; corridor walking and progressive activity from POD3	Routine nursing with encouragement for early ambulation	Post-op 7 d lung function; 6-month QLICP-LU; complications	Improved FEV1, FVC and FEV1/FVC; improved QoL; complications lower but not statistically significant
Min, 2024 (47)	China	40/40	64.72/64.51	26/23; 65.0%/57.5%	NSCLC; elderly patients	Thoracoscopic radical lung cancer surgery	No preoperative chemo/radiotherapy	Early pulmonary rehabilitation within 24 h: upright sitting, diaphragmatic/pursed-lip breathing, incentive spirometry, cough/sputum training, ACBT, upper-limb training and ambulation	Routine rehabilitation nursing	POD3–5 pain; discharge FVC, FEV1, 6MWD, QLQ-C30	Reduced postoperative pain; higher FVC, FEV1, 6MWD and QoL at discharge

ACBT, active cycle of breathing technique; ADL, activities of daily living; C, control group; d, day(s); ERAS, enhanced recovery after surgery; FEV1, forced expiratory volume in 1 second; FVC, forced vital capacity; FTS, fast-track surgery; I, intervention group; LOS, length of hospital stay; NR, not reported; NSCLC, non-small cell lung cancer; POD, postoperative day; QoL, quality of life; RoB 2, revised Cochrane risk-of-bias tool for randomized trials; VATS, video-assisted thoracoscopic surgery; VC, vital capacity; VT, tidal volume; 6MWD, 6-minute walking distance; 6MWT, 6-minute walk test.

### 3.3 Quality assessment of the included studies

**Table 2** presents the risk-of-bias assessment of the 23 included studies using the ROB 2 tool. Overall, none of the studies were judged to be at low risk of bias. Four studies were rated as having a high overall risk of bias, mainly because of high risk in the randomization process, while the remaining 19 studies were judged as having some concerns. For the randomization process, five studies were rated as low risk, four as high risk, and the others as having some concerns. All studies were judged as having some concerns regarding deviations from intended interventions. Most studies had low risk for missing outcome data, but concerns remained in several studies. In addition, most studies were rated as having some concerns in the measurement of outcomes and selection of the reported results, largely because blinding and prespecified reporting information were insufficiently described. The GRADE assessment results are presented in **Table 3**. The evidence for the incidence of complications was rated as high quality, while the evidence for lung function and 6MWD was considered very low quality due to concerns regarding inconsistency.

**Table 2:** Risk of bias assessment using the Cochrane RoB 2 tool.

Study	D1: randomization process	D2: deviations from intended interventions	D3: missing outcome data	D4: measurement of the outcome	D5: selection of the reported result	Overall RoB 2 judgement
Arbane et al., (26)	Low risk	Some concerns	Some concerns	Some concerns	Some concerns	Some concerns
Brocki et al., (27)	Low risk	Some concerns	Low risk	Some concerns	Low risk	Some concerns
Chen et al., (10)	High risk	Some concerns	Low risk	Some concerns	Some concerns	High risk
Chen et al., (29)	High risk	Some concerns	Low risk	Some concerns	Some concerns	High risk
Deng et al., (30)	Some concerns	Some concerns	Low risk	Some concerns	Some concerns	Some concerns
Ding et al., (31)	High risk	Some concerns	Low risk	Some concerns	Some concerns	High risk
Feng et al., (32)	High risk	Some concerns	Low risk	Some concerns	Some concerns	High risk
Granger et al., (54)	Low risk	Some concerns	Some concerns	Low risk	Low risk	Some concerns
Jonsson et al., (33)	Low risk	Some concerns	Some concerns	Low risk	Low risk	Some concerns
Jonsson et al., (34)	Low risk	Some concerns	Some concerns	Low risk	Low risk	Some concerns
Quan et al., (35)	Some concerns	Some concerns	Low risk	Some concerns	Some concerns	Some concerns
Shi et al., (36)	Some concerns	Some concerns	Low risk	Some concerns	Some concerns	Some concerns
Wang, (37)	Some concerns	Some concerns	Low risk	Some concerns	Some concerns	Some concerns
Wang et al., (38)	Some concerns	Some concerns	Some concerns	Some concerns	Some concerns	Some concerns
Wu et al., (39)	Some concerns	Some concerns	Low risk	Some concerns	Some concerns	Some concerns
Yan et al., (40)	Some concerns	Some concerns	Low risk	Some concerns	Some concerns	Some concerns
Yang, (41)	Some concerns	Some concerns	Low risk	Some concerns	Some concerns	Some concerns
Yang et al., (42)	Some concerns	Some concerns	Low risk	Some concerns	Some concerns	Some concerns
Yin, (43)	Some concerns	Some concerns	Low risk	Some concerns	Some concerns	Some concerns
Zhang et al., (44)	Some concerns	Some concerns	Low risk	Some concerns	Some concerns	Some concerns
Zhu, (45)	Some concerns	Some concerns	Low risk	Some concerns	Some concerns	Some concerns
Yunting, (46)	Some concerns	Some concerns	Low risk	Some concerns	Some concerns	Some concerns
Min, (47)	Some concerns	Some concerns	Low risk	Some concerns	Some concerns	Some concerns

**Table 3:** Grading of recommendation, assessment, development, and evaluation (GRADE) assessment.

Outcome	Studies, n	Quality assessment					Patients (I/C)	Effect, mean difference (95% CI)	Quality
		Risk of bias	Inconsistency	Indirectness	Imprecision	Publication bias			
Lung Function	7	No	Very Serious	No	Serious	Undetected	397/399	0.73 (0.38 to 1.09)	Very low
Duration of chest tube retention	17	No	Very Serious	No	No	Undetected	456/455	-1.28 (-1.54, -1.03)	Low
LOS	13	No	Very Serious	No	No	Undetected	702/703	-3.22 (-4.05, -2.38)	Low
Incidence of complications	15	No	No	No	No	Undetected	666/664	0.33 (0.25, 0.44)^*^	High
6MWD	5	No	Very Serious	No	Serious	Undetected	144/146	61.85 (20.60, 103.10)	Very low

I, intervention group; C, control group; LOS, length of hospital stay; 6MWD, 6-minute walking distance. *Risk ratio (RR) value.

### 3.4 Effectiveness of early activity intervention among patients with lung cancer

#### 3.4.1 Lung function

**Figure 2:**
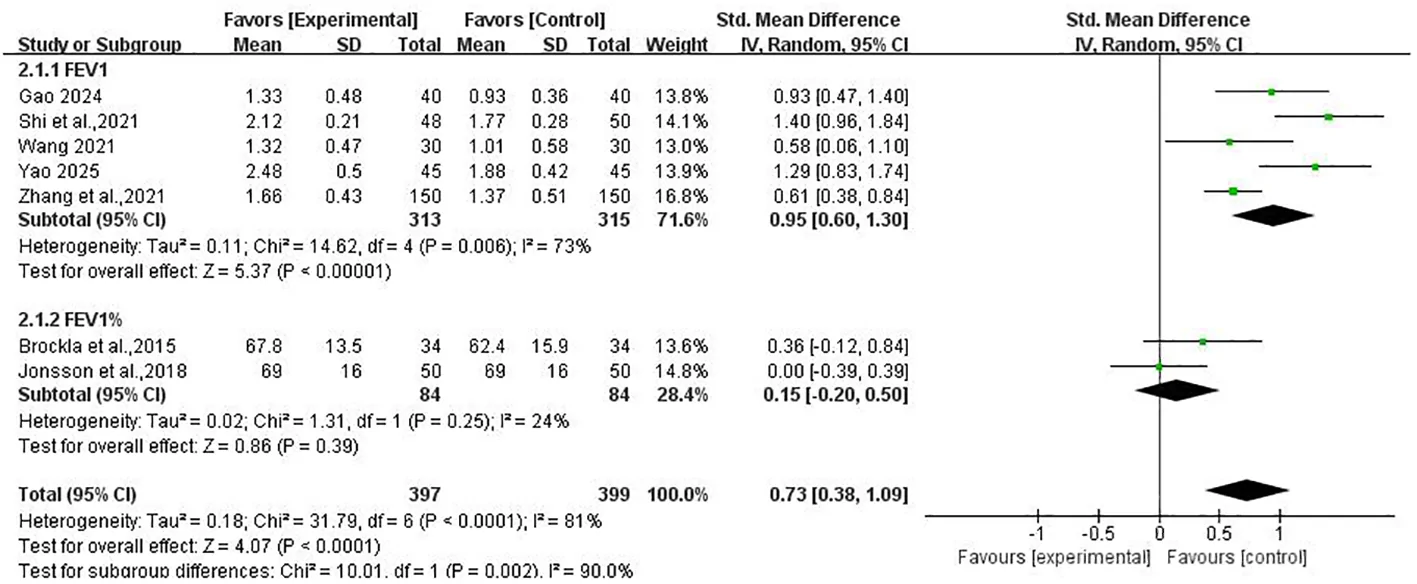
Forest plot of lung function.

Among the included studies, only seven trials reported the effect of early activity on lung function in patients with lung cancer (27, 33, 36, 37, 44, 46, 47). Specifically, five studies (36, 37, 44, 46, 47) used forced expiratory volume in the first second (FEV1), while two used FEV1%. Because the measurement units varied, the SMD was selected as the effect measure. Given the significant heterogeneity among studies (*I*^2^ = 81%, *p*<0.001), subgroup analyses were performed. The results showed that early activity significantly improved FEV1 compared with the control group (SMD = 0.95, 95% CI: 0.60 to 1.30, *p* < 0.001). In contrast, the pooled effect for FEV1% was not statistically significant (SMD = 0.15, 95% CI: –0.20 to 0.50, *p* = 0.39) (**Figure 2**).

#### 3.4.2 Duration of chest tube retention

**Figure 3:**
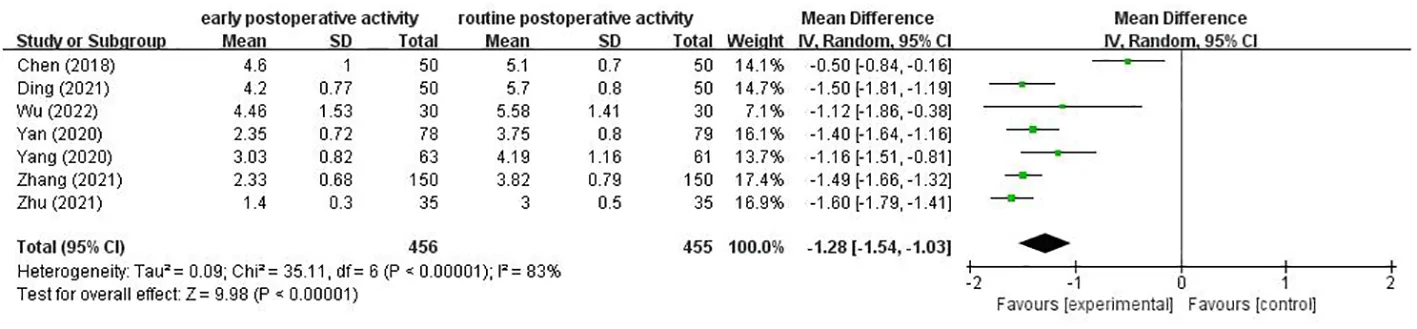
Forest plot of chest tube duration.

Seven studies (28, 31, 39, 40, 42, 44, 45) analyzed the effect of early postoperative activity on chest tube duration, involving 911 patients (456 in the intervention group and 455 in the control group). As pre-planned, a random-effects model was used for the analysis. Substantial statistical heterogeneity was observed among the included studies (*I*^2^ = 83%, *p*<0.001). Meta-analysis showed that the early postoperative activity group had a significant shorter chest tube duration than the routine activity group (MD=-1.28, 95% CI: -1.54 to -1.03, *p*<0.001) (**Figure 3**). To explore potential sources of heterogeneity, subgroup analyses were performed based on the timing of intervention initiation (6h vs. 24h) and surgical method (thoracoscopic vs. not mentioned), but no significant subgroup differences were observed (**Supplementary Appendix 3, Supplementary e-Figures 1, 2**).

#### 3.4.3 LOS

**Figure 4:**
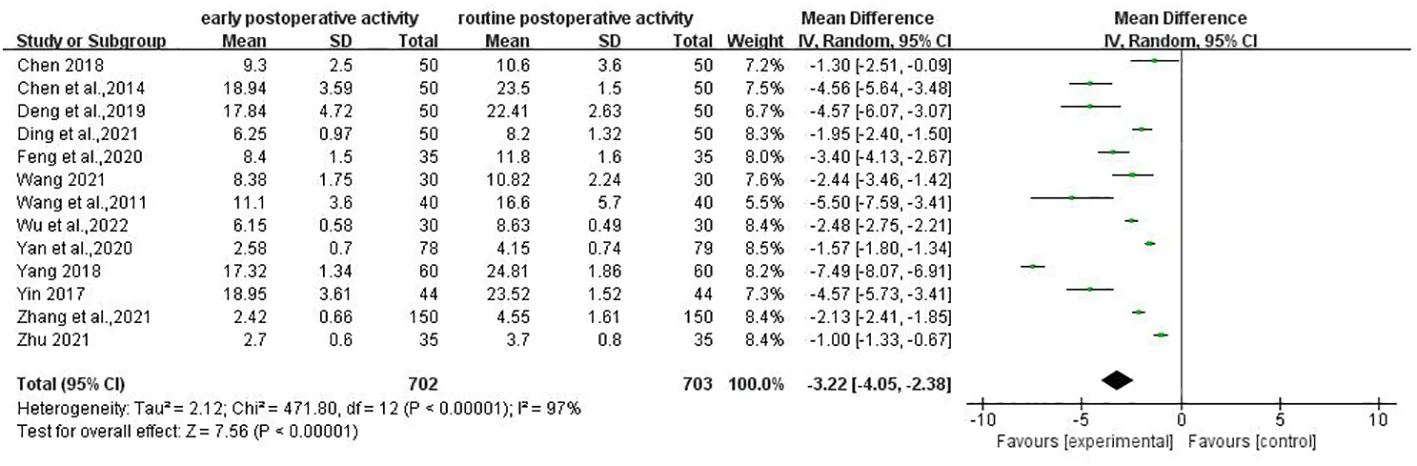
Forest plot of LOS.

Thirteen studies (28–32, 37–41, 43–45) analyzed the effect of early postoperative activity on LOS. Due to the presence of substantial heterogeneity (*I*^2^ = 97%, *p*<0.001), a random effects model was employed. Meta-analysis showed that early postoperative activity significantly reduced patients’ LOS by an average of 3.22 days. (MD=-3.22, 95% CI: -4.05 to -2.38, *p*<0.001) (**Figure 4**).

Subgroup analyses were conducted to explore potential sources of heterogeneity. When stratified by the timing of early activity initiation, both the 6-hour subgroup (MD = –2.20, 95% CI: –2.75 to –1.65) and the 24-hour subgroup (MD = –4.03, 95% CI: –6.02 to –2.04) showed significant reductions in LOS, although the effect appeared more pronounced in the 24-hour subgroup.

Stratification by surgical approach indicated that studies involving thoracoscopic surgery (MD = –2.20, 95% CI: –2.75 to –1.65), those that did not specify the approach (MD = –4.03, 95% CI: –6.02 to –2.04), and those including both open and thoracoscopic surgery (MD = –5.50, 95% CI: –7.59 to –3.41) all favored early activity. Significant subgroup differences were observed (*p* = 0.003).

Additionally, analysis by type of intervention revealed that walking alone (MD = –4.76, 95% CI: –5.72 to –3.80), walking combined with breathing exercises (MD = –2.13, 95% CI: –2.41 to –1.85), and integrated intervention programs (defined as multi-component protocols combining three or more distinct modalities, such as early ambulation and limb resistance exercises) (MD = –3.05, 95% CI: –4.10 to –2.00) were all effective. Furthermore, significant subgroup differences (*p*<0.001) were found (**Supplementary Appendix 3, Supplementary e-Figures 3–5**).

Sensitivity analysis was performed by sequentially excluding individual studies and reanalyzing the data. The *I²* values did not change significantly, and the pooled effect sizes remained consistent with those of the original meta-analysis. These findings indicate that the results are robust and not driven by any single study. However, further high-quality trials are warranted to strengthen these conclusions.

#### 3.4.4 Incidence of complications

**Figure 5:**
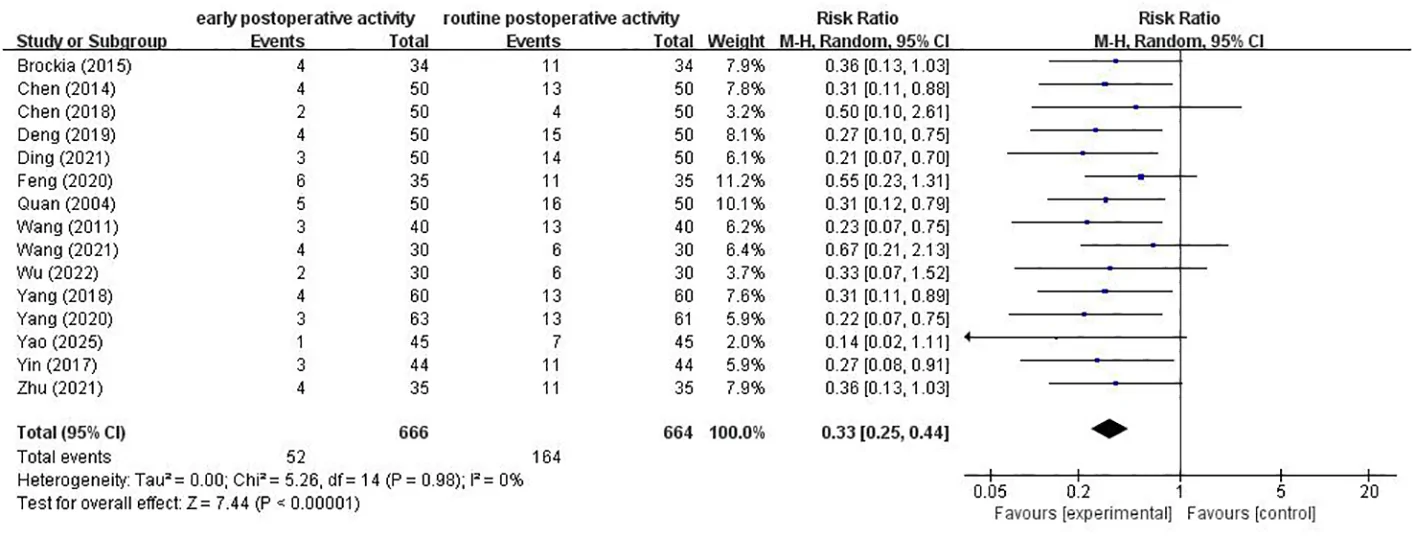
Forest plot of incidence of complications.

Fifteen studies (27–32, 35, 37–39, 41–43, 45, 46) reported postoperative complication rates, specifically investigating the incidence of pneumonia and atelectasis. The result showed a significant difference between the intervention and control groups, confirming that early postoperative activity significantly reduced the incidence of pulmonary complications (RR = 0.33, 95% CI: 0.25 to 0.44, *p*<0.001) (**Figure 5**).

#### 3.4.5 6MWD

The 6MWD is an essential indicator of exercise capacity in patients with lung cancer. Five studies reported the 6MWD following the interventions (22, 27, 31, 37, 38). Subgroup analyses were performed based on the duration and setting of the intervention: short-term interventions (solely in-hospital early activity) and long-term interventions (in-hospital early activity combined with post-discharge home exercise).

**Figure 6:**
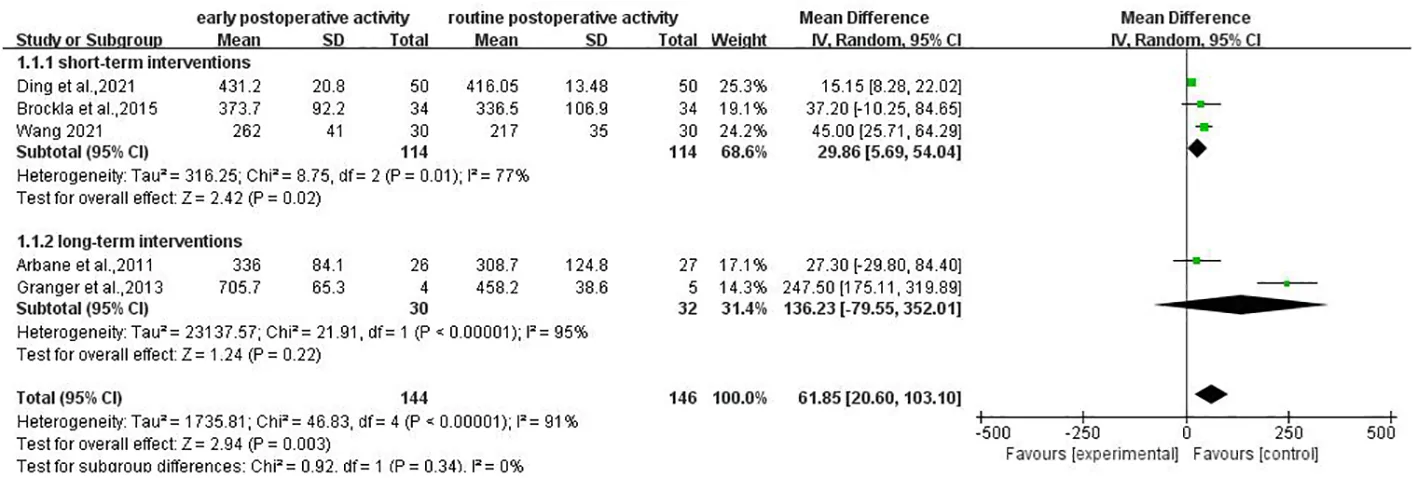
Forest plot of 6MWD.

For the subgroup receiving in-hospital early activity alone, the difference in 6MWD between the two groups was statistically significant (*p* = 0.02, *I*^2^ = 77%). However, when in-hospital early activity combined with home exercise, the difference in 6MWD between the two groups was not statistically significant (*p* = 0.22, *I*^2^ = 95%) (**Figure 6**).

### 3.5 Publication bias

**Figure 7:**
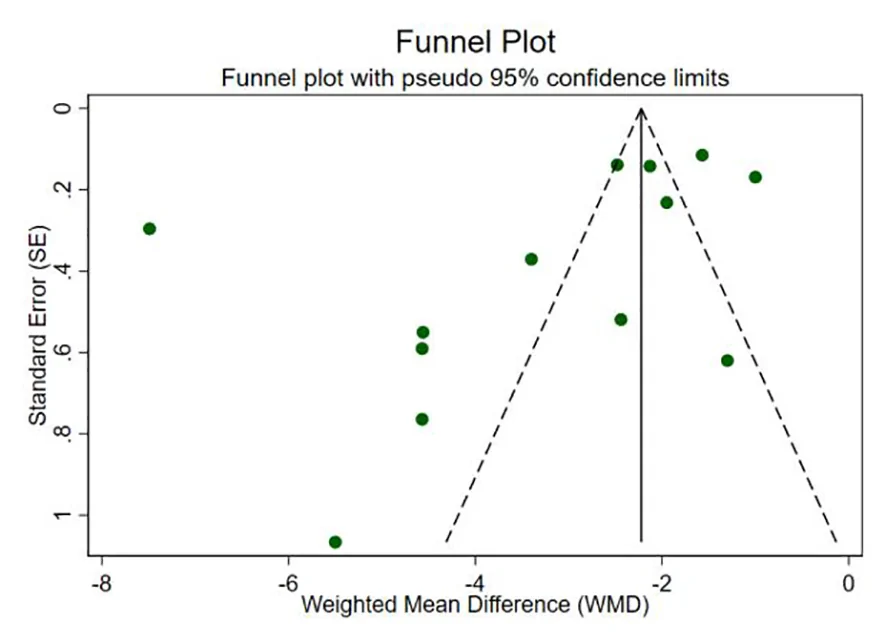
Funnel plots of LOS and incidence of complications.

Publication bias was not detected through the visual evaluation of funnel plots (**Figure 7**) and this was further confirmed by Egger’s test for both LOS (*p=*0.092) and incidence of complications (*p* = 0.397).

## 4 Discussion

### 4.1 Main findings

To determine the effect of early activity on the prognosis of postoperative lung cancer patients, this study analyzed 23 RCTs with 2,234 patients. The main findings were that early activity reduced chest tube duration and LOS, reduced the incidence of pulmonary complications, and improved lung function and exercise capacity, effectively improving patient prognosis.

The results of this study are not entirely consistent with previous research (48–50). Specifically, previous literature has reported mixed or inconclusive effects regarding postoperative interventions. For instance, while Ahn et al. demonstrated that structured inpatient exercise significantly reduced LOS, their study was limited to colon cancer patients whose primary recovery metric was bowel motility. More notably, a recent meta-analysis by Xu et al. investigating lung cancer concluded that while preoperative interventions were effective, short-term postoperative rehabilitation alone did not significantly reduce postoperative pulmonary complications or LOS. Similarly, the systematic review by Castelino et al. found conflicting and insufficient evidence to confirm that early mobilization protocols significantly improved outcomes or reduced complications across general abdominal and thoracic surgeries. These discrepancies may be explained by several factors. First, there is heterogeneity in outcome definitions (e.g., complications are classified differently across studies). Second, there is variation in the operational definition of “early activity”, ranging from ambulation alone to combined respiratory and strength training. Third, differences exist in study populations, including baseline functional status and comorbidities. Finally, geographic distribution may play a role, as 16 of the 23 studies in our review were conducted in Asian countries, where clinical practice patterns and perioperative care pathways may differ from those in Western countries. Taken together, these contextual differences likely contribute to the variability in reported outcomes across studies and should be considered when interpreting our findings.

### 4.2 Effects on lung function

Although early postoperative activity improves lung function, the effects are not uniform across different indicators. Subgroup analysis revealed that early activity significantly improved absolute FEV1, indicating a meaningful enhancement in ventilatory capacity; however, no statistically significant improvement was observed for FEV1%. The lack of effect on FEV1% may be attributed to inherent variability in the prediction equations used, alongside differences in individual baseline characteristics (e.g., age, sex, and height). These confounding factors limit cross-study comparability and consequently reduce the sensitivity of this metric in detecting subtle changes during postoperative rehabilitation.

Notably, one study (36) reported a particularly large effect size for FEV1, which was greater than those of the other included studies. This unusually large effect may be explained by the specific intervention design, which focused on systematic early respiratory function training. Compared with the simple ambulation protocols employed in other trials, this targeted approach may have led to greater short-term improvements in FEV1. Additionally, the study was conducted in a single center with high intervention adherence, which could have amplified the observed benefit. While these findings support the potential value of structured respiratory training, they also contributed substantially to the statistical heterogeneity in the pooled analysis and should be interpreted with caution.

### 4.3 Effects on chest tube duration and LOS

Early activity after lung cancer surgery reduces chest tube duration and LOS. Chest tube duration is universally recognized as an essential indicator of postoperative recovery in patients with lung cancer. Prolonged retention can lead to complications such as infection, subcutaneous emphysema, and pulmonary atelectasis (51). The results of this study showed that early activity after lung cancer surgery significantly shortened chest tube duration and decreased LOS, which was crucial for promoting rapid postoperative recovery.

Subgroup analyses further indicated that early initiation within 6 hours was associated with particularly consistent benefits and minimal heterogeneity, while significant effects were also observed at 24 hours and in thoracoscopic cases, though with greater variability between studies. Moreover, subgroup analyses of LOS further revealed that both very early (within 6 hours) and early (within 24 hours) mobilization significantly reduced LOS, with more pronounced effects observed in the 24-hour subgroup. Benefits were also consistent across different surgical approaches (thoracoscopic, open plus thoracoscopic, or not reported) and across various intervention types (walking alone, walking plus breathing exercises, or integrated programs), though the magnitude of effect varied. These findings suggest that both the timing and the content of early activity, as well as the surgical approach, may influence recovery outcomes and partly explain the heterogeneity among studies.

Physiologically, early postoperative physical activity programs can facilitate the expulsion of fluid and gas from the chest cavity and promote lung re-expansion, thereby shortening chest tube duration. However, from a psychological perspective, getting out of bed within 24 hours poses a considerable challenge. Patients often express a strong preference for bed rest, driven by conventional beliefs, incisional pain, and fears of accidentally dislodging their chest drainage tubes during movement (52). To overcome these barriers, clinical practice must adopt a comprehensive approach. Nurses should provide robust preoperative education detailing the specific exercises, frequency, and overall importance of early physical activity to enhance compliance. Simultaneously, raising awareness among families, implementing effective pain management strategies, and equipping hospital wards with appropriate mobility aids are essential steps to alleviate patient fears and facilitate safe, effective early postoperative mobilization (53).

### 4.4 Effects on pulmonary complications

Although surgical resection remains the primary curative treatment for lung cancer, postoperative pulmonary complications such as pneumonia, arrhythmia, and pulmonary atelectasis are not uncommon and affect patient prognosis. The results of this study show that early activity after lung cancer surgery can effectively reduce the incidence of these postoperative complications, which aligns with the findings reported by Zhang (44). Specifically, multi-component interventions that combined early ambulation with structured respiratory exercises proved to be highly effective. The potential mechanisms contributing to these positive effects likely include the enhanced clearance of airway secretions, improved ventilation-perfusion matching, and the recruitment of collapsed alveoli, which directly counteract the respiratory impairments caused by surgical trauma and anesthesia. Clinically, these findings highlight the necessity of moving beyond traditional bed rest by implementing standardized, early mobilization protocols that pair breathing exercises with walking within the first 24 hours. Future research should focus on determining the optimal dose-response relationship, evaluating the specific intensity, frequency, and duration of early activity required to maximize the reduction of these postoperative complications.

### 4.5 Effects on exercise capacity

Exercise capacity in this review was mainly assessed using the 6MWD. Overall, the included studies suggested that early activity may help preserve or improve postoperative exercise capacity; however, the findings were not completely consistent across studies. Some trials reported improvements in 6MWD after early activity or rehabilitation-based interventions, whereas others found no significant between-group difference. This inconsistency may be explained by differences in intervention content, intensity, supervision, duration, follow-up time, baseline physical function, and surgical approach.

The 6MWD is a simple and widely used measure of functional exercise capacity, reflecting the patient’s ability to perform daily physical activity. A change in 6MWD is clinically relevant because previous lung cancer research has suggested that the minimal important difference for deterioration in 6MWD is approximately 22–42 m, or about 9.5% (54). Therefore, improvements or smaller postoperative declines in 6MWD may indicate better functional recovery, although statistical significance alone should not be interpreted as clinical significance.

Based on the included studies, interventions that may be more likely to improve exercise capacity include multicomponent programs combining early ambulation, respiratory training, progressive walking, limb or resistance exercises, and continued supervised or semi-supervised rehabilitation after discharge. In contrast, brief low-intensity mobilization during hospitalization alone may be insufficient to produce a measurable improvement in 6MWD, especially when the intervention duration is short or patients have relatively good baseline exercise capacity.

Clinically, these findings suggest that early activity should not be limited to simply encouraging patients to get out of bed. Instead, postoperative activity should be individualized, progressive, supervised when possible, and integrated with respiratory and strength-based components. Future studies should standardize the reporting of intervention dose, intensity, supervision, adherence, and follow-up duration, and should report whether changes in 6MWD exceed clinically meaningful thresholds.

## 5 Strengths and limitations

The primary strength of this meta-analysis is its focus on early activity in the context of ERAS. Additionally, to comprehensively evaluate the impact of early mobilization on patient prognosis following lung cancer surgery, we searched both English and Chinese electronic databases and analyzed five outcomes. Moreover, our review was conducted strictly following the PRISMA guideline.

However, this meta-analysis has certain limitations. First, the majority of the included studies were single-center trials with relatively small sample sizes, which may have resulted in an overestimation of effect sizes. Most studies were conducted in China, and clinical practices, perioperative care pathways, discharge policies, and patient characteristics may differ across countries and healthcare systems. Therefore, the generalizability of these findings to other clinical settings and populations may be limited. In addition, some outcomes were supported by only a small number of studies. For example, the analysis of exercise capacity, assessed using 6MWD, included only five studies with limited sample sizes. Therefore, the findings for 6MWD should be interpreted with particular caution and should be considered exploratory rather than definitive.

Second, the potential impact of performance and detection bias should be considered. Because early activity interventions are difficult to blind, most included studies did not blind participants, healthcare providers, or outcome assessors. This may have influenced patient behavior, provider encouragement, co-interventions, and outcome assessment, particularly for subjective or judgement-based outcomes such as complications, pain, quality of life, and functional recovery. Even relatively objective outcomes, such as LOS and chest tube duration, may still be affected by clinical decision-making and institutional practice. Therefore, the observed benefits may have been overestimated and should be interpreted with caution.

Third, significant clinical and statistical heterogeneity was observed among the included studies, particularly for LOS, chest tube duration, and 6MWD. Importantly, the definition and implementation of “early activity” varied substantially across studies, which may have influenced the pooled results. Although all interventions were initiated within the first 24 postoperative hours, the intervention components varied considerably, ranging from simple ambulation to combined respiratory training, strength training, and aerobic exercise. Additional variability arose from differences in sample size, baseline patient characteristics, surgical techniques, perioperative ERAS pathways, intervention duration, supervision, adherence, and outcome measurement methods. For LOS and chest tube duration, heterogeneity may also have been influenced by institutional discharge policies, chest tube removal criteria, and local clinical practice patterns. For 6MWD, the small number of included studies and differences in follow-up duration and post-discharge exercise protocols further limited the certainty of the findings. Although subgroup analyses and random-effects models were used to explore or account for heterogeneity, these approaches could not fully explain the observed variability. Therefore, the pooled estimates for these outcomes should be interpreted cautiously, especially regarding the magnitude of effect rather than only the direction of benefit. Additionally, although we used two independent reviewers for study selection, data extraction, and risk of bias assessment to improve the reliability and consistency of the review process and to minimize reviewer errors, the pooled results should nevertheless be interpreted with caution given the overall risk of bias and substantial heterogeneity among the included studies.

Future research should focus on large-scale, multicenter RCTs with more rigorous designs and standardized early mobilization protocols to validate the clinical benefits of this approach.

## 6 Conclusion

In conclusion, this systematic review demonstrates that early activity significantly reduces the incidence of pulmonary complications, shortens chest tube duration, and decreases LOS in patients recovering from lung cancer surgery. Furthermore, early activity also improves lung function, particularly absolute FEV1, while no significant effect was observed for FEV1%.

Regarding exercise capacity, limited evidence from five studies suggests that short-term early in-hospital activity may be associated with improved 6MWD, whereas the combination of early in-hospital activity and home exercise programs did not show a significant benefit and was accompanied by substantial heterogeneity. Therefore, conclusions regarding exercise capacity should be interpreted with caution. These results suggest that while in-hospital mobilization is beneficial, the long-term effects of combined programs remain uncertain, largely due to variability in intervention protocols and patient adherence. Future large-scale, multicenter RCTs with standardized definitions and protocols for early activity are needed to provide more robust evidence and to clarify both the isolated and long-term effects of these interventions in this patient population.

## Data Availability

The original contributions presented in the study are included in the article/**Supplementary Material**. Further inquiries can be directed to the corresponding author.
