# Supplementary material for: The effect of early activity on postoperative patients with lung cancer: a systematic review and meta-analysis
Source: Front Oncol. 2026 Aug 20;16:1840866. doi: 10.3389/fonc.2026.1840866 (PMC13537943; doi:10.3389/fonc.2026.1840866)
Supplement: Supplementary file 1 [file DataSheet1.docx]

**Appendix 3 Subgroup analyses of outcomes**

e-Figure 1. Subgroup analysis by time of early postoperative activity on duration of chest tube retention

e-Figure 2. Subgroup analysis by surgical method on chest tube retention

e-Figure 3. Subgroup analysis by time of early postoperative activity on length of stay in hospital

e-Figure 4. Subgroup analysis by surgical method on length of stay in hospital

e-Figure 5. Subgroup analysis by intervention type on length of stay in hospital
