# Supplementary material for: The effect of early activity on postoperative patients with lung cancer: a systematic review and meta-analysis
Source: Front Oncol. 2026 Aug 20;16:1840866. doi: 10.3389/fonc.2026.1840866 (PMC13537943; doi:10.3389/fonc.2026.1840866)
Supplement: Supplementary file 2 [file Table1.docx]

**Appendix 1 Search strategy**

| **Patients** | **Intervention** | **Comparator** | **Outcome** |
| --- | --- | --- | --- |
| Lung Neoplasms | Measures for early activity, including breathing exercises, early mobility, aerobic exercise and muscle strength training | No applicable | Duration of chest tube retention; Lung function; LOS; Incidence of complications; 6MWD |

Table 1. The search terms

| **Database** | **Search strategy** | **Results** |
| --- | --- | --- |
| Pubmed | ("Lung Neoplasms"[MESH] OR "Pulmonary Neoplasms"[TA] OR "Neoplasms, Lung"[TA] OR "Lung Neoplasm"[TA] OR "Pulmonary Neoplasm"[TA] OR "Lung Cancer"[TA] OR "Lung Cancers"[TA] OR "Pulmonary Cancer"[TA] OR "Pulmonary Cancers"[TA] OR "Cancer of Lung"[TA] OR "Cancer of the Lung"[TA] OR "Lung malignancy"[TA]) AND ("Exercise"[MESH] OR "Exercises"[TA] OR "Physical Activity"[TA] OR "Activities, Physical"[TA] OR "Activity, Physical"[TA] OR "Physical Activities"[TA] OR "Exercise, Physical"[TA] OR "Exercises, Physical"[TA] OR "Physical Exercise"[TA] OR "Physical Exercises"[TA] OR "Exercise, Aerobic"[TA] OR "Aerobic Exercise"[TA] OR "Aerobic Exercises"[TA] OR "Exercises, Aerobic"[TA] OR "Exercise Training"[TA] OR "Exercise Trainings"[TA] OR "Training, Exercise"[TA] OR "Exercise Therapy"[TA] OR "Therapy, Exercise"[TA] OR "Exercise Therapies"[TA] OR "Therapies, Exercise"[TA] OR "Rehabilitation Exercise"[TA] OR "Exercise, Rehabilitation"[TA] OR "Exercises, Rehabilitation"[TA] OR "Rehabilitation Exercises"[TA] OR "Remedial Exercise"[TA] OR "Exercise, Remedial"[TA] OR "Remedial Exercises"[TA] OR "Resistance Training"[TA] OR "Training, Resistance"[TA] OR "Training, Strength"[TA] OR "Strength Training"[TA] OR "Walking"[TA] OR "Rehabilitation"[TA] OR "Pulmonary rehabilitation"[TA] OR "inspiratory muscle training"[TA] OR "respiratory muscle training"[TA] OR "Breathing Exercise"[TA] OR "Breathing Exercises"[TA] OR "Exercise, Breathing"[TA] OR "Respiratory Muscle Training"[TA] OR "Muscle Training, Respiratory"[TA] OR "Training, Respiratory Muscle"[TA] OR "Expiratory muscle training"[TA] OR "Physical activity"[TA] OR "Upper limb activities"[TA] OR "Upper limb movement"[TA] OR "Upper limb movements"[TA] OR "Upper Extremity Activities"[TA] OR "Lower limb activities"[TA] OR "Lower limb mobility"[TA] OR "Lower limb movement"[TA] OR "Lower Extremity Activities"[TA] OR "shoulder motion"[TA] OR "early rehabilitation"[TA]) | 1540 |
| Embase | #1 ('lung neoplasms' OR 'lung neoplasm' OR 'pulmonary neoplasms' OR 'neoplasms, lung' OR 'neoplasm, lung' OR 'neoplasms, pulmonary' OR 'neoplasm, pulmonary' OR 'pulmonary neoplasm' OR 'lung cancer' OR 'cancer, lung' OR 'cancers, lung' OR 'lung cancers' OR 'pulmonary cancer' OR 'cancer, pulmonary' OR 'cancers, pulmonary' OR 'pulmonary cancers' OR 'cancer of the lung' OR 'cancer of lung' OR 'lung malignancy'):ab,ti  #2 ('Exercise' OR 'Exercises' OR 'Physical Activity' OR 'Activities, Physical' OR 'Activity, Physical' OR 'Physical Activities' OR 'Exercise, Physical' OR 'Exercises, Physical' OR 'Physical Exercise' OR 'Physical Exercises' OR 'Exercise, Aerobic' OR 'Aerobic Exercise' OR 'Aerobic Exercises' OR 'Exercises, Aerobic' OR 'Exercise Training' OR 'Exercise Trainings' OR 'Training, Exercise' OR 'Exercise Therapy' OR 'Therapy, Exercise' OR 'Exercise Therapies' OR 'Therapies, Exercise' OR 'Rehabilitation Exercise' OR 'Exercise, Rehabilitation' OR 'Exercises, Rehabilitation' OR 'Rehabilitation Exercises' OR 'Remedial Exercise' OR 'Exercise, Remedial' OR 'Remedial Exercises' OR 'Resistance Training' OR 'Training, Resistance' OR 'Training, Strength' OR 'Strength Training'):ab,ti  #3 #1 AND #2 | 2487 |
| WOS | TI=("Lung Neoplasms" OR "Pulmonary Neoplasms" OR "Neoplasms, Lung" OR "Lung Neoplasm" OR "Pulmonary Neoplasm" OR "Lung Cancer" OR "Lung Cancers" OR "Pulmonary Cancer" OR "Pulmonary Cancers" OR "Cancer of Lung" OR "Cancer of the Lung") AND TI=("Exercise" OR "Exercises" OR "Physical Activity" OR "Activities, Physical" OR "Activity, Physical" OR "Physical Activities" OR "Exercise, Physical" OR "Exercises, Physical" OR "Physical Exercise" OR "Physical Exercises" OR "Exercise, Aerobic" OR "Aerobic Exercise" OR "Aerobic Exercises" OR "Exercises, Aerobic" OR "Exercise Training" OR "Exercise Trainings" OR "Training, Exercise" OR "Exercise Therapy" OR "Therapy, Exercise" OR "Exercise Therapies" OR "Therapies, Exercise" OR "Rehabilitation Exercise" OR "Exercise, Rehabilitation" OR "Exercises, Rehabilitation" OR "Rehabilitation Exercises" OR "Remedial Exercise" OR "Exercise, Remedial" OR "Remedial Exercises" OR "Resistance Training") | 536 |
| Cochrane | #1 "Exercise":ti,ab,kw OR "Exercises":ti,ab,kw OR "Physical Activity":ti,ab,kw OR "Activities, Physical":ti,ab,kw OR "Activity, Physical":ti,ab,kw OR "Physical Activities":ti,ab,kw OR "Exercise, Physical":ti,ab,kw OR "Exercises, Physical":ti,ab,kw OR "Physical Exercise":ti,ab,kw OR "Physical Exercises":ti,ab,kw OR "Exercise, Aerobic":ti,ab,kw OR "Aerobic Exercise":ti,ab,kw OR "Aerobic Exercises":ti,ab,kw OR "Exercises, Aerobic":ti,ab,kw OR "Exercise Training":ti,ab,kw OR "Exercise Trainings":ti,ab,kw OR "Training, Exercise":ti,ab,kw OR "Exercise Therapy":ti,ab,kw OR "Therapy, Exercise":ti,ab,kw OR "Exercise Therapies":ti,ab,kw OR "Therapies, Exercise":ti,ab,kw OR "Rehabilitation Exercise":ti,ab,kw OR "Exercise, Rehabilitation":ti,ab,kw OR "Exercises, Rehabilitation":ti,ab,kw OR "Rehabilitation Exercises":ti,ab,kw OR "Remedial Exercise":ti,ab,kw OR "Exercise, Remedial":ti,ab,kw OR "Remedial Exercises":ti,ab,kw OR "Resistance Training":ti,ab,kw  #2 "Lung Neoplasms":ti,ab,kw OR "Pulmonary Neoplasms":ti,ab,kw OR "Neoplasms, Lung":ti,ab,kw OR "Lung Neoplasm":ti,ab,kw OR "Pulmonary Neoplasm":ti,ab,kw OR "Lung Cancer":ti,ab,kw OR "Lung Cancers":ti,ab,kw OR "Pulmonary Cancer":ti,ab,kw OR "Pulmonary Cancers":ti,ab,kw OR "Cancer of Lung":ti,ab,kw OR "Cancer of the Lung":ti,ab,kw "Lung malignancy":ti,ab,kw  #3 #1 AND #2 | 832 |
| CINAHL | ((TI 'Lung Neoplasms' OR TI 'Pulmonary Neoplasms' OR TI 'Neoplasms, Lung' OR TI 'Lung Neoplasm' OR TI 'Pulmonary Neoplasm' OR TI 'Lung Cancer' OR TI 'Lung Cancers' OR TI 'Pulmonary Cancer' OR TI 'Pulmonary Cancers' OR TI 'Cancer of Lung' OR TI 'Cancer of the Lung' OR TI 'Lung malignancy')) AND ((TI 'Exercise' OR TI 'Exercises' OR TI 'Physical Activity' OR TI 'Activities, Physical' OR TI 'Activity, Physical' OR TI 'Physical Activities' OR TI 'Exercise, Physical' OR TI 'Exercises, Physical' OR TI 'Physical Exercise' OR TI 'Physical Exercises' OR TI 'Exercise, Aerobic' OR TI 'Aerobic Exercise' OR TI 'Aerobic Exercises' OR TI 'Exercises, Aerobic' OR TI 'Exercise Training' OR TI 'Exercise Trainings' OR TI 'Training, Exercise' OR TI 'Exercise Therapy' OR TI 'Therapy, Exercise' OR TI 'Exercise Therapies' OR TI 'Therapies, Exercise' OR TI 'Rehabilitation Exercise' OR TI 'Exercise, Rehabilitation' OR TI 'Exercises, Rehabilitation' OR TI 'Rehabilitation Exercises' OR TI 'Remedial Exercise' OR TI 'Exercise, Remedial' OR TI 'Remedial Exercises' OR TI 'Resistance Training')) | 653 |
| CNKI | SU %= (肺肿瘤 + 癌, 非小细胞肺 + 非小细胞肺癌 + 肺癌 + 肺恶性肿瘤 + 原发性支气管癌 + 支气管癌 + 非小细胞肺肿瘤 + 非小细胞瘤 + 非小细胞性肺癌) AND SU %= (早期活动 + 早日下床活动 + 早期下床活动 + 术后早期活动 + 早期离床活动 + 术后早期运动 + 早期运动 + 早期康复 + 体力活动 + 早期锻炼 + 早期训练) | 270 |
| Wanfang | 主题:("[肺肿瘤](javascript:toDetail('cross','D008175','120');)" or "[癌, 非小细胞肺](javascript:toDetail('cross','D002289','120');)" or "非小细胞肺癌" or "肺癌" or "肺恶性肿瘤" or "原发性支气管癌" or "支气管癌" or "非小细胞肺肿瘤" or "非小细胞瘤" or "非小细胞性肺癌") and 主题:("早期活动" or "早日下床活动" or "早期下床活动" or "术后早期活动" or "早期离床活动" or "术后早期运动" or "早期康复" or "体力活动" or "早期锻炼" or "早期训练" or "早期运动") | 367 |
| VIP | U = (肺肿瘤 OR [癌, 非小细胞肺](javascript:toDetail('cross','D002289','120');) OR 非小细胞肺癌 OR 肺恶性肿瘤 OR 原发性支气管癌 OR 支气管癌 OR 非小细胞肺肿瘤 OR 非小细胞瘤 OR 非小细胞性肺癌) AND (早期活动 OR 早日下床活动 OR 早期下床活动 OR 术后早期活动 OR 早期离床活动 OR 术后早期运动 OR 早期运动 OR 早期康复 OR 体力活动 OR 早期锻炼 OR 早期训练) | 217 |
| CBM | (("早期活动"[常用字段] OR "早日下床活动"[常用字段] OR "术后早期活动"[常用字段] OR "早期离床活动"[常用字段] OR "术后早期运动"[常用字段] OR "早期运动"[常用字段] OR "早期康复"[常用字段] OR "体力活动"[常用字段] OR "早期锻炼"[常用字段] OR "早期训练"[常用字段])) AND ("肺肿瘤"[常用字段] OR "癌, 非小细胞肺"[常用字段] OR "非小细胞肺癌"[常用字段] OR "肺恶性肿瘤"[常用字段] OR "原发性支气管癌"[常用字段] OR "支气管癌"[常用字段] OR "非小细胞肺肿瘤"[常用字段] OR "非小细胞瘤"[常用字段] OR "非小细胞性肺癌"[常用字段]) | 239 |

Table 2. The search results across the databases
