# Supplementary material for: The effect of early activity on postoperative patients with lung cancer: a systematic review and meta-analysis
Source: Front Oncol. 2026 Aug 20;16:1840866. doi: 10.3389/fonc.2026.1840866 (PMC13537943; doi:10.3389/fonc.2026.1840866)
Supplement: Supplementary file 3 [file Table2.docx]

**Appendix 2 Sensitivity analysis of LOS**

| **Sensitivity analysis of LOS** | | | | |
| --- | --- | --- | --- | --- |
| **Omitting the study** | **Mean Difference(95% CI)** | ***P*_for overall effect_** | ***I*^2^**  **(%)** | ***P*_for Heterogeneity_** |
| Brockia et al.,2015 | -0.30(-2.92,2.32) | ＜0.001 | 97 | ＜0.001 |
| Chen 2018 | -1.30(-2.51,-0.09) | ＜0.001 | 97 | ＜0.001 |
| Chen et al.,2014 | -4.56(-5.64,-3.48) | ＜0.001 | 97 | ＜0.001 |
| Deng et al.,2019 | -4.57(-6.07,-3.07) | ＜0.001 | 97 | ＜0.001 |
| Ding et al.,2021 | -1.95(-2.40,-1.50) | ＜0.001 | 97 | ＜0.001 |
| Feng et al.,2020 | -3.40(-4.13,-2.67) | ＜0.001 | 97 | ＜0.001 |
| Wang 2021 | -2.44(-3.46,-1.42) | ＜0.001 | 97 | ＜0.001 |
| Wang et al.,2011 | -5.50(-7.59,-3.41) | ＜0.001 | 97 | ＜0.001 |
| Wu et al.,2022 | -2.48(-2.75,-2.21) | ＜0.001 | 97 | ＜0.001 |
| Yan et al.,2020 | -1.57(-1.80,-1.34) | ＜0.001 | 97 | ＜0.001 |
| Yang 2018 | -7.49(-8.07,-6.91) | ＜0.001 | 92 | ＜0.001 |
| Yin 2017 | -4.57(-5.73,-3.41) | ＜0.001 | 97 | ＜0.001 |
| Zhang et al.,2021 | -2.13(-2.41,-1.85) | ＜0.001 | 97 | ＜0.001 |
| Zhu 2021 | -1.00(-1.33,-0.67) | ＜0.001 | 97 | ＜0.001 |

Table1: Sensitivity analysis of LOS
